# Comprehensive analysis of differences of N^6^-methyladenosine of lncRNAs between atrazine-induced and normal *Xenopus laevis* testis

**DOI:** 10.1186/s41021-021-00223-0

**Published:** 2021-11-06

**Authors:** Xuejie Qi, Xiao Geng, Juan Zhang, Binpeng Qu, Xin Zhang, Qiang Jia, Wenhui Yin, Cunxiang Bo, Yan Liu, Hao Li, Linlin Sai, Mingming Han, Cheng Peng

**Affiliations:** 1 Department of Toxicology, Shandong Academy of Occupational Health and Occupational Medicine, Shandong First Medical University & Shandong Academy of Medical Sciences, 18877 Jingshi Road, Lixia District, Ji’nan, Shandong China; 2 Shandong Medical College, Ji’nan, Shandong China; 3 School of Laboratory Animal & Shandong Laboratory Animal Center, Shandong First Medical University & Shandong Academy of Medical Sciences, Ji’nan, Shandong China; 4 Queensland Alliance for Environmental Health Sciences (QAEHS), The University of Queensland, Brisbane, Australia

**Keywords:** RNA methylation, M^6^A, LncRNA, Amphibious, Atrazine

## Abstract

**Background:**

Increasing evidence suggested N^6^-methyladenosine (m^6^A) modification is crucial for male germline development. However, m^6^A modification of lncRNAs gains a little attention in amphibians in recent years. *Xenopus laevis* (*X. laevis*) was chosen to be an ideal model organism for testing environmental endocrine disrupting chemicals (EDCs) exposure and resultant effects. Atrazine (AZ) as an endocrine disrupt can effect development of testis in amphibians. Our previous study revealed that m^6^A is a highly conserved modification across the species.

**Results:**

The results of m^6^A sequences showed that m^6^A-methylated lncRNAs enriched in intergenic region in testes of *X. laevis*. We further examined the differential expression of lncRNAs m^6^A sites in testes of AZ-exposed and compared with that in animals from control group. The results indicated that up to 198 differentially methylated m^6^A sites were detected within 188 lncRNAs, in which 89 significantly up-methylated sites and 109 significantly down-methylated sites. Data from KEGG pathway analysis indicated that AZ-affected lncRNAs m^6^A sites were mainly involved in 10 pathways in which 3 mutual pathways were found in the result of differentially m^6^A-methylated mRNAs.

**Conclusions:**

These findings suggested that differentially m^6^A-methylated lncRNAs and these 3 pathways may act on regulatory roles in abnormal testis development of AZ-exposed *X. laevis*. This study for the first time provides insights into the profile of lncRNAs m^6^A modifications in amphibian species.

**Supplementary Information:**

The online version contains supplementary material available at 10.1186/s41021-021-00223-0.

## Introduction

RNA modifications play crucial roles in gene expression [1]. As the most universal form of post-transcriptional RNA modifications, N^6^-methyladenosine (m^6^A) modification has become a new research area in epigenetic [2]. Recent studies have shown that the m^6^A modification modulates the function of the RNA molecule in multiple ways through its novel functions [3, 4]. The regulatory association between RNA m^6^A modification and spermatogenic function has been discovered [5]. Cumulative studies have found that knockout of RNA m^6^A regulators in the testis leads to abnormal metabolism of the target RNAs, which eventually causes spermatogenetic disorders and infertility [6]. The m^6^A modification is found in different species of RNA, including tRNA, mRNA, rRNA, and long non-coding RNAs (lncRNAs) [7, 8]. In addition, the m^6^A modification can affects many properties of RNA, including gene translation [9], splicing [10], and long non-coding RNA-mediated gene silencing [11].

LncRNAs are non-coding RNAs comprising more than 200 nucleotides without protein coding function and engaging in diverse biological processes across every branch of life [12]. Increasing evidence indicetates that lncRNAs play an important role in regulating multiple processes of gene expression [13]. Studies have also found that regulation of lncRNAs can affect mRNA transcription, splicing, translation and stability [14]. It has been widely recognized that dysregulated lncRNAs play an important part in many diseases [15]. In recent years, m^6^A modification of lncRNAs gains great attention and this modification has shown to control mammalian gene expression [16]. To our knowledge, the profile of m^6^A modification of lncRNAs in amphibians remain to be explored.

Atrazine (2-chloro-4-ethylamino-6-isopropylamino-s-triazine, AZ) is an EDCs used extensively as an herbicide worldwide [17, 18]. AZ has been reported that it can cause endocrine disruption in mammals, birds, reptiles, and amphibians by affecting normal reproductive function and development in these organisms [19, 20]. *Xenopus laevis* (*X. laevis*) is a kind of amphibian widely used as an ideal model organism for testing EDCs exposure [21]. Recently, AZ has been shown to cause demasculinization and complete feminization in male *X. laevis* [22]. In our previous studies, we investigated biological response of *X. laevis* exposed to AZ (0.1, 1, 10 or 100 μg/L) for 90 days in the water environment. We found that AZ induced the reduction of gonad weight and gonado somatic index of male *X. laevis*. Meanwhile, AZ induced histological changes in testes of the frogs from all of AZ treatments including irregular shape of seminiferous lobules and large empty spaces [23]. However, the mechanism of AZ-induced abnormal development of male *X. laevis* is unclear. Therefore, it is necessary to explore the potential changes of m^6^A modification of lncRNAs which maybe play an important role in the abnormal testis of male AZ-exposed *X. laevis*.

In general, mRNA m^6^A is enriched around the stop codon and 3′ UTR in mammals, hypothesized to contribute towards the control of transcript stability and translation [24]. However, in our previous results, the m^6^A peak observed clearly enriched in the start codon and stop codon in *X. laevis* [25]. In contrast to mRNAs, m^6^A residues in lncRNAs are distributed along the whole body of transcripts and are more concentrated in the lncRNAs undergoing alternative splicing. Furthermore, Dominissini et al. also identified that m^6^A in exonic regions was preferentially found in longer exons of 400 nucleotides or more [26]. LncRNAs are defined as seven types of transcripts such as including included lincRNA, antisense, processed transcript, sense intronic, 3 prime overlapping ncRNA, sense overlapping, and macro lncRNA [27].

Here, we first analysed the profile of m^6^A modification of lncRNAs in *X. laevis* and dysregulated m^6^A methylation of lncRNAs in the AZ-exposed male *X. laevis.* Then, we predicted classification function and involved signaling pathways of dysregulated m^6^A methylation of lncRNAs in AZ-exposed male *X. laevis*. Our data will provide the basis for future studies of m^6^A methylation of lncRNAs about function and biological significance in amphibians and the insightful information of the abnormal testis development in AZ-exposed male *X. laevis.*

## Methods

### Ethic approval

All animal experiments were performed in accordance with relevant guidelines and regulations. All experiments were complied with the “Principles of Animal Care”. The protocol was assessed and approved by the Committee on the Ethics of Animal Experiments of Shandong Academy of Occupational Health and Occupational Medicine.

### Sample animals

The adult male and female *X. laevis* were purchased from the Chinese Academy of Sciences (Beijing, China) and natural mated to produce offspring. UV-treated and carbon-filtered laboratory freshwater was used for the acclimatization of frogs in the laboratory and for all subsequent exposures. The *X. laevis* were kept at an average water temperature of 22 ± 2 °C at pH 7.5, under 12 h light and 12 h dark cycle. Tadpoles were fed fairy shrimp (*Artemia nauplii*) eggs in a young age daily and pork liver three times per week ad libitum when the tadpoles completed metamorphosis.

At Nieuwkoop-Faber (NF) stage 47 (13 d post-hatch), mixed sex tadpoles (*n* = 320) from one adult pairing were randomly divided into two groups. AZ (purity of 97%) obtained from Sigma (Chemical Co., USA) dissolved in solvent vehicle DMSO (0.01%). The tadpoles were exposed to AZ at dosages of 100 μg/L for 180 days. The control tadpoles were treated with 0.01% DMSO only. Test solutions were refreshed by 50% replacement every 48 h. Animals were observed daily for monitoring morphological changes and health status [23]. The *X. laevis* were sacrificed after being exposed to AZ for 180 days. The exposure time is based on the developmental characters of male *X. laevis*. The testis tissues were collected and weight, and then stored at − 80 °C immediately for further analysis.

### LncRNAs preparation

For each group, at least three biological replicates were run [28]. Three testes from controls and three ones from 100 μg/L AZ-treated groups were selected randomly for lncRNAs analysis. Then, total RNA of tissue was extracted using TRIzol reagent (Invitrogen Corporation, CA, USA). The concentration and purity of RNA were evaluated by NanoDrop® ND-2000 spectrometer (Thermo, Waltham, MA, USA). The integrity of RNA was determined by denaturing gel electrophoresis. RNA samples were further purified and converted to double-stranded cDNA for microarray analysis which was conducted according to Agilent® *Xenopus* 4 × 44 K Gene Expression Microarrays protocols.

### lncRNAs m^6^A MeRIP sequencing

M^6^A of lncRNAs was sequenced by MeRIP sequencing using the latest Illumina HiSeq sequencer. Briefly, fragmented RNA was incubated with anti-m^6^ A polyclonal antibody (Synaptic Systems, 202,003) in IPP buffer for 2 h at 4 °C and the mixture was immune precipitated. Then, bound RNA was eluted from the beads in buffer and then extracted by following the manufacturer’s instruction. Both the input sample without immune precipitation and the m^6^A IP samples were subjected to 150 bp paired-end sequencing on Illumina HiSeq sequencer. Paired-end reads were harvested and were quality controlled by Q30. Detailed methods were described in our previous study [25].

### Data analysis

After sequencing, quality control of the paired-end reads was performed with Q30, which was subjected to 3’adaptor trimming and low quality reads removing to generate clean reads by Cutadapt software (v1.9.3). Firstly, clean reads of all libraries were aligned to reference genome using bowtie 2 [29] software and mapped to genome by hisat 2 software (v2.04) [30]. Methylated sites on lncRNAs (methylated sites with a score (− 10*log10, *P*-value) of > 3) were identified by MACS software. Differentially m^6^A-methylated sites on lncRNAs were detected by diffReps and the identified sites overlapping with exons of lncRNAs were chosen for further analysis. Pathway enrichment analysis was used to maps genes to Kyoto Encyclopedia of Genes and Genomes (KEGG) pathways.

## Results

### Overview of m^6^A sites within lncRNAs in the testes of control and AZ-exposed *X. laevis*

**Fig. 1:**
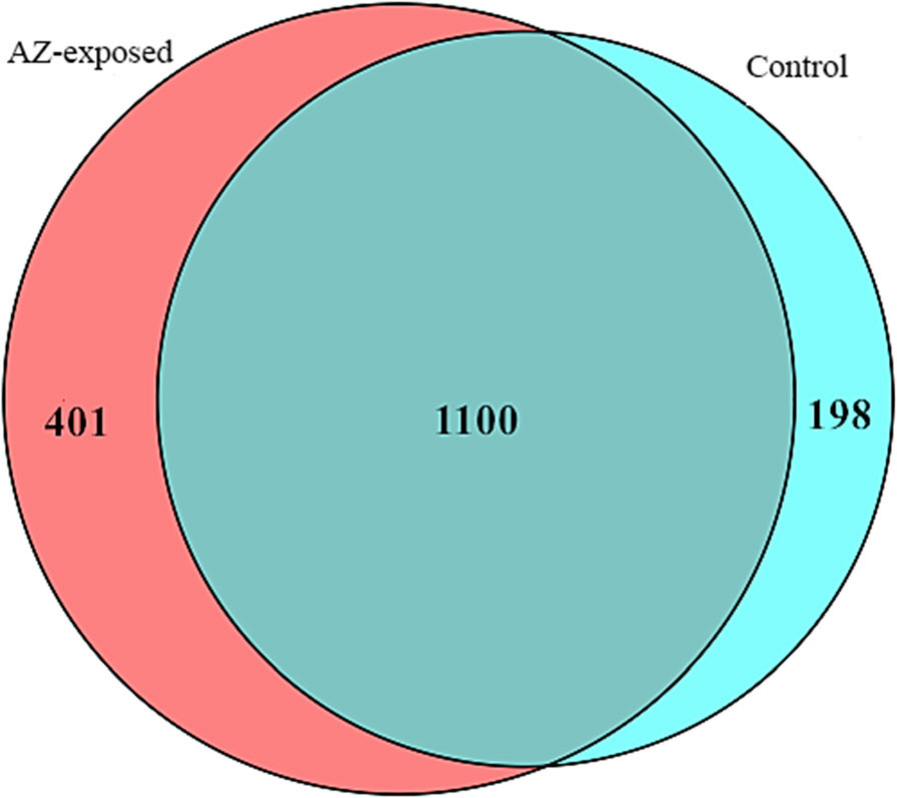
Venn diagram showing the overlap of m^6^A peaks within lncRNAs in AZ-exposed and control groups

MeRIP-seq analysis of lncRNAs derived from the testes of *X. laevis* revealed that there were 1298 m^6^A peaks among 908 lncRNAs in control group. While 1501 m^6^A peaks among 1055 lncRNAs were detected in the testes of AZ exposed *X. laevis*. Importantly, 1100 m^6^A recurrent sites were consistently detected in controls and AZ-exposed groups (Fig. 1).

### Distribution profiles of m^6^A-methylated lncRNAs

**Fig. 2:**
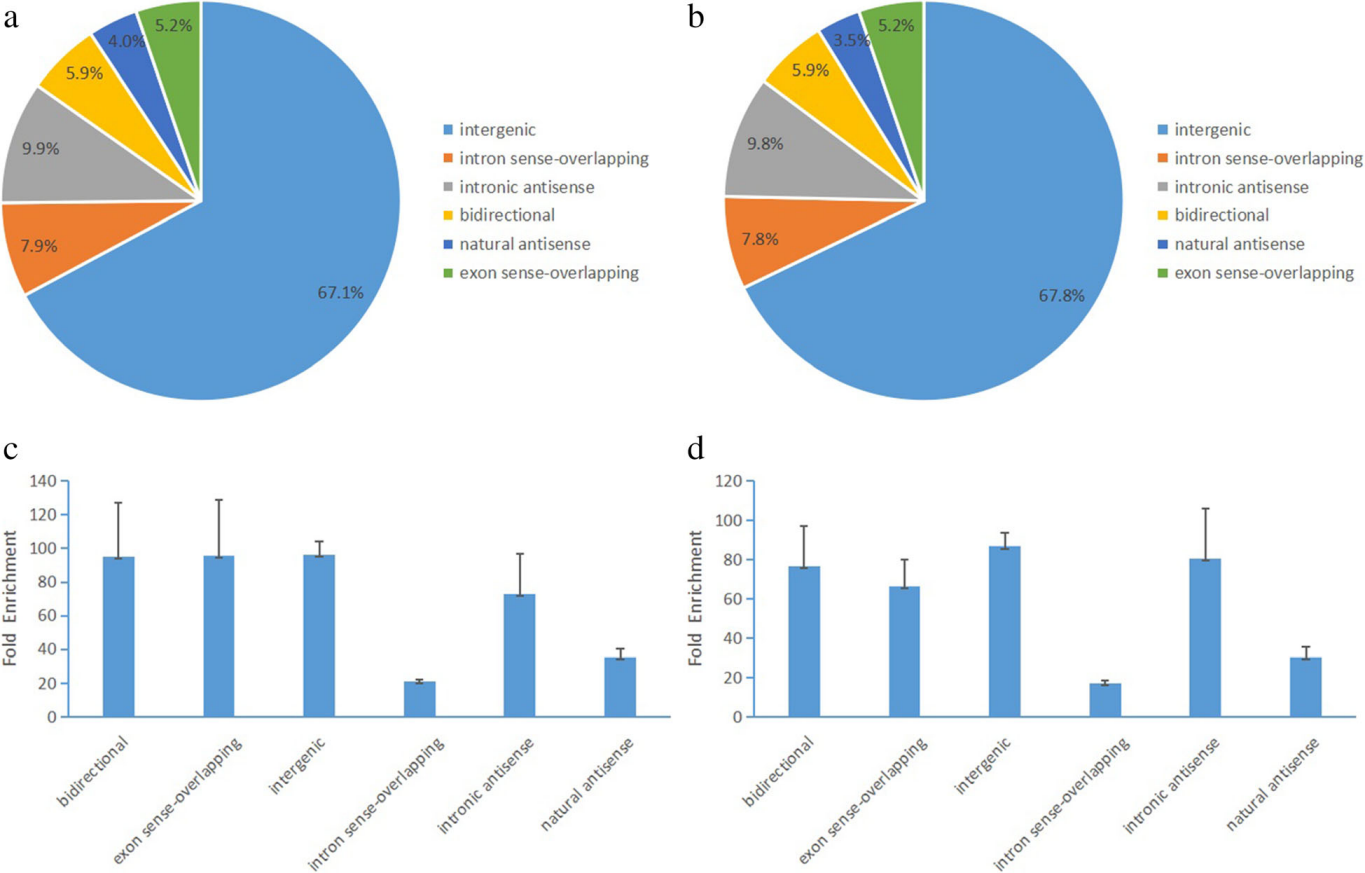
Overview the distribution positions of m^6^A-methylated lncRNAs in the testes of *X. laevis* from control and AZ-exposed groups. **a**: Pie charts showing the percentage of the distribution positions of m^6^A-methylated lncRNAs relatived to its mRNA in control group. **b**: Pie charts showing the percentage of the distribution positions of m^6^A-methylated lncRNAs in AZ-exposed group. **c**: Distributions of mean fold enrichment of m^6^A-methylated lncRNAs in six segments in control group. **d**: Distributions of mean fold enrichment of m^6^A-methylated lncRNAs in six segments in AZ-exposed group. Error bars represent the standard error of the mean. The mean fold enrichment in the intergenic segments was the largest both in the control and AZ-exposed group with lower standard error of the mean

To further analyze the distribution profiles of m^6^A-methylated lncRNAs, these distribution positions of modified lncRNAs were categorized into 6 groups: bidirectional, exon sense-overlapping, intergenic, intron sense-overlapping, intron sense-overlapping, natural antisense. Particularly, we found that the most m^6^A-methylated lncRNAs are highly enriched in intergenic region (67.1% in control and 67.8% in AZ-exposed groups) (Fig. 2a and b). In the controls, the distribution positions of m^6^A-methylated lncRNAs had a highly fold enrichment in bidirectional (95.24%), exon sense-overlapping (95.74%) and intergenic regions (96.15%). Meanwhile, in AZ-exposed groups, the distribution positions of m^6^A-methylated lncRNAs had a highly fold enrichment in intergenic (86.65%), intronic antisense (80.47%) and bidirectional (76.88%) (Fig. 2c and d).

### Differentially m^6^A modification sites of lncRNAs in *X. laevis* exposed to 100 μg/L AZ

The results showed that 198 differentially methylated m^6^A sites were detected among 188 lncRNAs, in which 89 significantly up-methylated sites and 109 significantly down-methylated sites (Table S1). The top ten up- and down-methylated m^6^A sites of lncRNAs with the highest fold change (FC) values were shown in Tables 1 and 2.

**Table 1:** The top ten up-methylated m^6^A sites of lncRNAs

Chromosome	txStart	txEnd	lncRNA	FC
NC_030726.1	167,623,498	167,623,720	LOC108707576	143.6
NC_030729.1	96,905,501	96,905,826	LOC108713215	140.5
NC_030729.1	42,037,587	42,037,701	LOC108712839	119.9
NC_030737.1	37,948,621	37,948,839	LOC108697950	107.9
NC_030726.1	78,976,541	78,976,920	LOC108708210	101.9
NC_030730.1	122,514,814	122,514,835	LOC108713571	99.0
NC_030727.1	93,713,360	93,713,391	LOC108708955	96.1
NC_030727.1	83,393,506	83,393,693	LOC108708939	90.1
NC_030724.1	53,570,461	53,570,705	LOC108699628	87.1
NC_030730.1	105,478,624	105,478,680	LOC108713666	87.0

txStart/txEnd: Start/end position of the differentially methylated RNA sites.

**Table 2:** The top ten down-methylated m^6^A sites of lncRNAs

Chromosome	txStart	txEnd	lncRNA	FC
NC_030730.1	56,714,441	56,715,000	LOC108713510	108.0
NC_030737.1	3,748,163	3,748,254	LOC108697148	93.6
NC_030727.1	30,334,141	30,334,313	LOC108709317	82.1
NC_030727.1	141,936,052	141,936,238	mmp8.S	79.6
NC_030741.1	23,682,418	23,682,540	LOC108702712	77.2
NC_030731.1	10,726,561	10,726,740	LOC108715064	77.2
NC_030733.1	59,942,967	59,943,104	LOC108717426	72.3
NC_030725.1	4,717,538	4,717,900	LOC108706487	71.8
NC_030736.1	79,214,125	79,214,280	LOC108695842	71.8
NC_030736.1	43,073,001	43,073,560	LOC108696274	70.0

txStart/txEnd: Start/end position of the differentially methylated RNA sites.

**Fig. 3:**
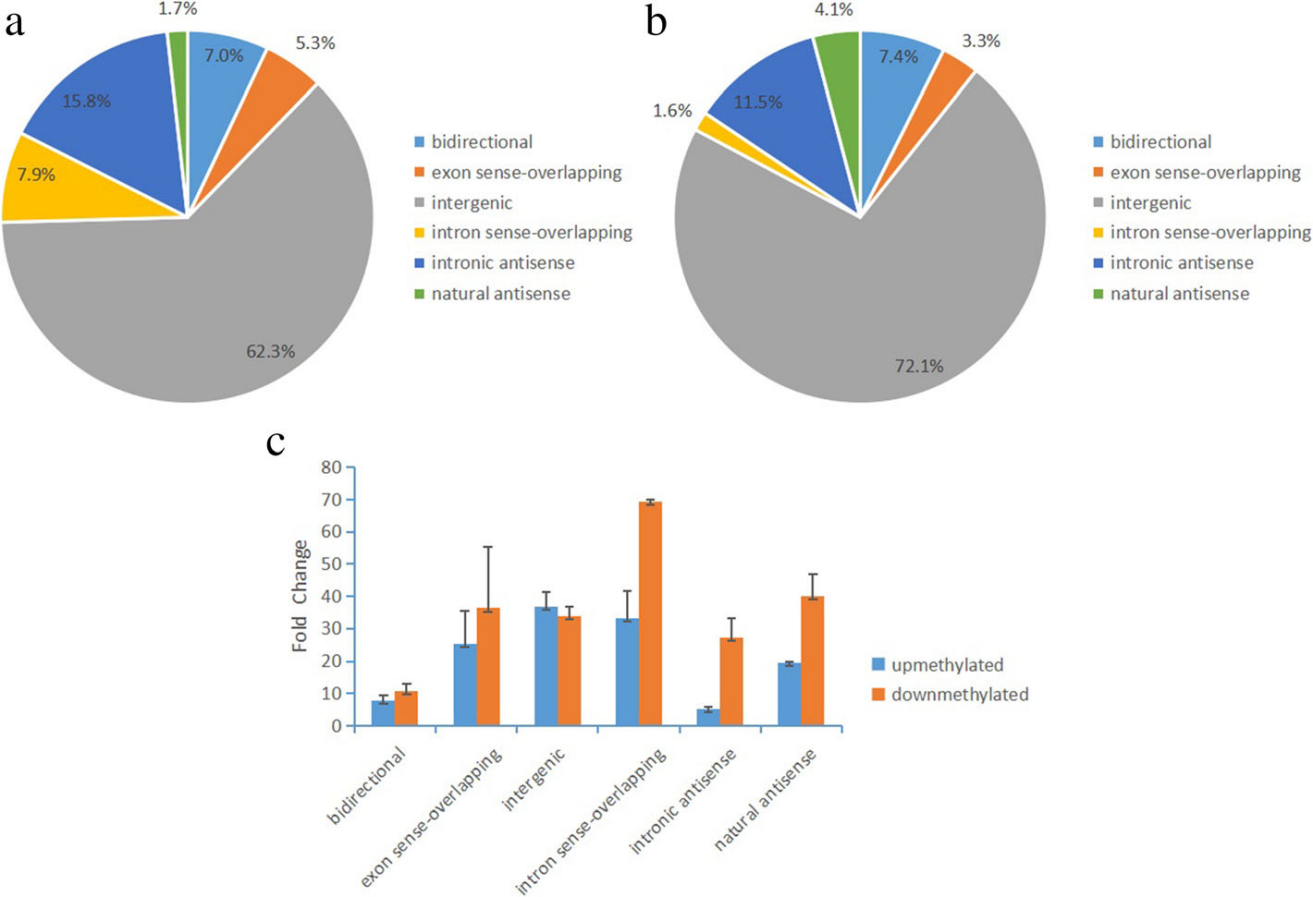
Distribution of differentially methylated m^6^A sites of lncRNAs. **a**: Pie charts showing the percentage of up-methylated m^6^A peaks in six segments. **b**: Pie charts showing the percentage of down-methylated m^6^A peaks in six segments. **c**: Statistics of meanof the distribution positions of m^6^A-methylated lncRNAs in six segments with up- and down-methylated sites. Error bars represent the standard error of the mean

Further analysis showed that according to the positional relationships of lncRNAs near the coding gene transcripts, most differentially methylated lncRNAs were assigned to intergenic (Fig. 3a and b). Besides, among the lncRNAs with up-methylated sites, those within the intergenic had the highest mean of FC. While among the lncRNAs with down-methylated sites, those within the intron sense-overlapping had the highest mean of FC (Fig. 3c).

### The enrichment pathways of differentially m^6^A-methylated lncRNAs-associated target genes by KEGG

**Fig. 4:**
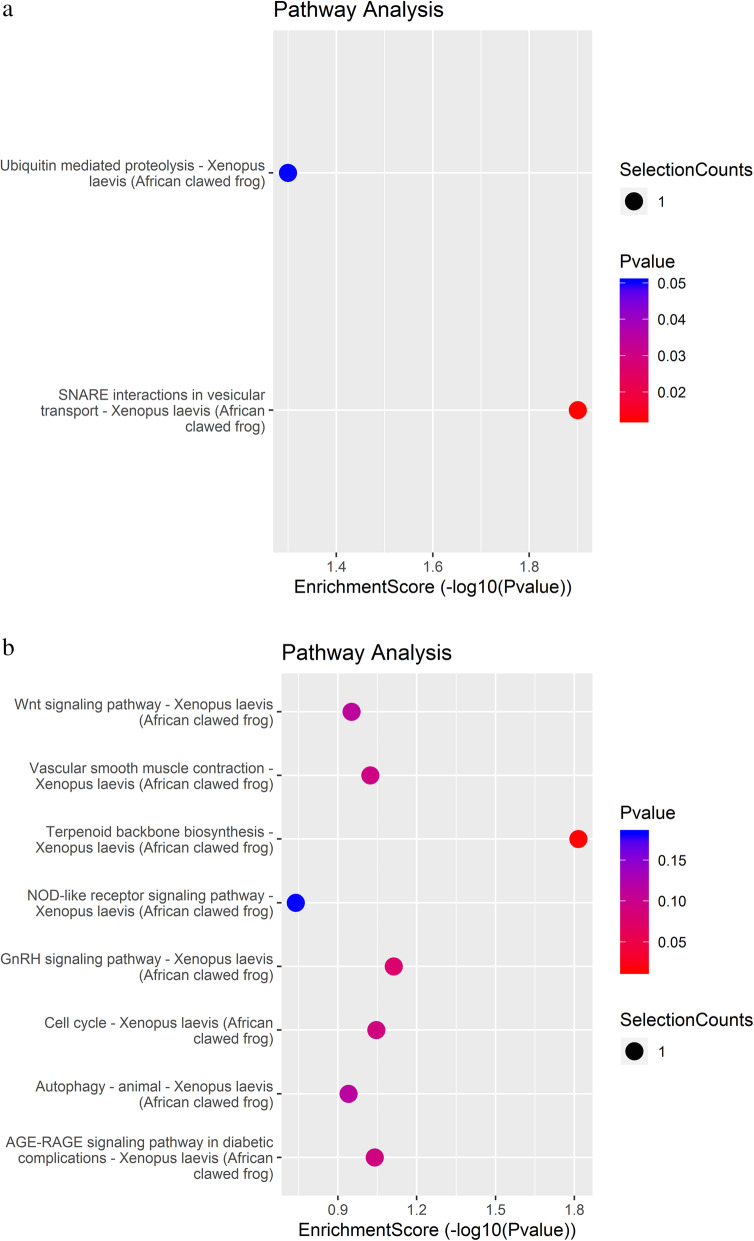
The annotated significant pathways targeted by the enrichment score of the differentially m^6^A-methylated (up-methylated (**a**) and down-methylated (**b**)) lncRNAs-related genes in testis of *X. laevis* exposed to 100 μg/L AZ. The horizontal axis is the -LogP (logarithm of *P*-value) for the pathway and the vertical axis is the pathway category

To further explore the roles of differentially m^6^A-methylated lncRNAs in the abnormal development of testis from AZ-exposed *X. laevis*, we performed KEGG pathway analysis of differentially m^6^A-methylated lncRNAs-related genes to look for the potential key pathways. The result of pathway analysis indicated that 2 pathways with highly enrichment score (−log10 (*P*-value)) were acquired in up-methylated sequencing data. The two signaling pathways, as “SNARE interactions in vesicular transport and Ubiquitin mediated proteolysis”, were shown in Fig. 4a. Meanwhile, 8 pathways were found in down-regulated sequencing data, including “Terpenoid backbone biosynthesis, GnRH signaling pathway, Cell cycle, AGE-RAGE signaling pathway in diabetic complications, Vascular smooth muscle contraction, Wnt signaling pathway, Autophagy-animal, NOD-like receptor signaling pathway” (Fig. 4b).

## Discussion

M^6^A modification is characterized by wide existence, unique distribution, and dynamic reversibility [31, 32]. It has also been found that enhancer RNAs, non-coding transcripts produced from enhancer regions are highly m^6^A modified [33]. M^6^A has been shown to be the abundant internal modification in eukaryotic mRNAs [34]. Emerging findings have shed light on the involvement of m^6^A modification of lncRNAs [35, 36]. A recent study showed that m^6^A methylation regulatory network regulates RNA processing and participates in various cellular biological processes, such as biological rhythm, immune modulation, fat metabolism, reproductive development [37]. Several studies showed that AZ could disrupt germ cell and Sertoli cell numbers. The *X. laevis* exposed to AZ has also been shown to reduce testicular volume and testosterone and induce testicular oogenesis [38–40]. Our results showed that AZ can elicit reproductive toxicity in developing male *X. laevis* in our previous study [23]. In addition, m^6^A, an epitranscriptomic mark regulating gene expression, plays essential roles during spermatogenesis [41]. Till date many potential lncRNAs have been reported in spermatogenesis and male infertility and they were identified to be important factors for spermatogenesis which could also be exploited as markers of male infertility [42]. To explore the m^6^A modification profile of lncRNAs in the testis of *X. laevis* and the changes of m^6^A modification of lncRNAs in AZ-exposed *X. laevis*, we examined the transcriptome-wide m^6^A modification of lncRNAs distribution in the testis of AZ*-*exposed *X. laevis.* Meanwhile, the changes of m^6^A modification of lncRNAs distribution were analyzed by exogenous stimulation.

The profiles of m^6^A modification of lncRNAs in mammals were identified in recent years, such as mouse, rat and human [43–46]. Meanwhile, large intergenic noncoding RNAs (lincRNAs) which are one class of lncRNAs transcribed from intergenic regions are defined [47].. Key roles for lincRNAs in certain biological processes are starting to emerge. LincRNAs are a novel class of gene regulators that function as signals, scaffolds, molecular decoys and mediators of long-range chromatin interactions [48]. The identification of specific lincRNAs has shown the importance of lincRNAs in developmental biology. For example, Yang et al. found that m^6^A modification of linc1281 mediated a competing endogenous RNA (ceRNA) model to regulate mouse embryonic stem cells differentiation [49]. In our study, the patterns of the m^6^A modification of lncRNAs were identified in *X. laevis*. Our results showed that the m^6^A-methylated lncRNAs mainly enriched in the intergenic region, namely m^6^A modification maily enriched in lincRNAs in *X. laevis*. Additionally, most of differentially expressed m^6^A modificationed also enriched in lincRNAs in AZ-exposed *X. laevis*. Our results suggested that m^6^A modification of lincRNAs may play a significant role in abnormal testis development of amphibian species.

Differentially m^6^A modification of lncRNAs were identified by comparing AZ-exposed testes of *X. laevis* to controls. Here, this result revealed a potential role of m^6^A modification sites of lncRNAs in testes of *X. laevis* induced by environmental agents such as AZ. Interestingly, we found lncRNA “XR_001933134” was up-regulated in the testis of AZ-treated *X. laevis* in our previous study, but the m^6^A modification of which was down-regulated [50]. The result showed that m^6^A modification may negatively regulate the expression of lncRNA “XR_001933134”. Wu et al. demonstrated that m^6^A modification of lncRNAs may increase lncRNA RP11 expression [35]. Ban et al. indicated that dysregulation of m^6^A modification might account for aberrant expression of LNCAROD in HNSCC [51]. Consequently, our results suggested that the negative regulatory relationship between m^6^A modification of lncRNAs and the expression of lncRNAs in abnormal testis development of *X. laevis* exposed to 100 μg/L AZ. Meanwhile, Liu et al. found that m^6^A modification participated in the upregulation of MALAT1 in renal fibrosis and m^6^A modification of lncRNA MALAT1 can increase its RNA stability in mammal [52]. Therefore, we predicted that m^6^A modification of lncRNAs may regulate their expression which involved in abnormal testis development of AZ-exposed *X. laevis*.

Up to now, no data has been reported about the pathway analysis of m^6^A-methylated lncRNA-associated target genes in AZ-treated *X. laevis*. Therefore, in the present study, we used KEGG pathway annotation method to analyze the m^6^A-methylated lncRNA-associated target genes in the testes of *X. laevis* exposed to100 μg/L AZ. The results of KEGG pathway analysis indicated that 10 pathways were involved in the current sequencing data.

In current study, the top one term was “SNARE interactions in vesicular transport” signaling pathway. SNARE (soluble N-ethylmaleimide-sensitive factor attachment protein receptor) proteins could drive vesicle fusion between endosomal compartments in eukaryotic cells [53]. SNARE proteins establish the core membrane fusion machinery of intracellular transport and intercellular communication, which contribute to cell growth, cell expansion, pathogen defense and homeostasis [54, 55]. Additionally, acrosome assembly in spermatogenesis and acrosome reaction in the interaction between sperm and oocyte are unique processes of vesicle synthesis, transportation and fusion, which are the basis of sperm fertilization [56, 57]. The previous study has also shown that SNARE syntaxin was associated with the acrosome in spermatids during sperm development in the testis [58]. Hence, we predicted that m^6^A-methylated lncRNAs included in SNARE interactions in vesicular transport signaling pathway may play an important role in the abnormal testis tissues of AZ-exposed *X. laevis*.

It is known that ubiquitin mediated proteolysis possesses many biological processes in controlling cell signaling, regulating cell proliferation, apoptosis, and immune responses [59, 60]. Ubiquitination is also a kind of the versatile cellular regulatory mechanisms and ubiquitin binds to protein playing a crucial role in substrate specificity [61]. In particular, several evidences have demonstrated that *X. laevis* offers the ability to generate soluble proteins, which capable to carry out the biochemical modifications of protein ubiquitylation [62]. Ubiquitylation usually occurs lysine residues and the residues could bond with ubmolecules and then target proteins for destruction [63]. Moreover, ubiquitin is highly expressed in mammalian gametes and embryos at any particular stage of development and ubiquitin ligasesare very active in the testis [64]. However, the study on ubiquitin mediated of gametogenesis in amphibian is sketchy. Our present study indicated that “ubiquitin mediated proteolysis pathway” regulated by m^6^A-methylated lncRNAs may involve in the abnormal testis development of *X. laevis* exposed to AZ.

Gonadotropin-releasing hormone (GnRH) was synthesized in hypothalamic neurons and binded to specific G-protein coupled receptors on the gonadotrope cell surface. It could regulate the biosynthesis and secretion of gonadotropin such as follicle-stimulating hormone (FSH) and luteinizing hormone (LH) which are required for the testis to produce bothmature sperm [65, 66]. Recent reports have shown that active immunization against GnRH could inhibit synthesis or secretion of gonadotropins, and thereby induced the termination of gametogenesis, inhibited reproductive behavior, and finally caused infertility of both male and female animals [67]. Moreover, orexin receptors type 1 (OX1R) was G protein-coupled receptors whose receptor expression was found in the pituitary of *X. laevis* [68]. The expression level was regulated by gonadal GnRH [65]. Therefore, m^6^A-methylated lncRNAs involved in “GnRH signaling pathway”may play an important role in damaged testis of AZ-exposed *X. laevis.*

Interestingly, “SNARE interactions in vesicular transport”, “NOD-like receptor signaling pathway” and “GnRH signaling pathway” were also found in the results of KEGG of differentially m^6^A-methylated mRNAs in *X. laevis* exposed to 100 μg/L AZ in our previous study [25]. The results showed that 3 mutual pathways may play important regulatory roles and possibly induce testes damage in AZ-exposed *X. laevis*.

## Conclusion

We examined the m^6^A modification profile of lncRNAs in testis tissues of *X. laevis* with and without treatment with 100 μg/L AZ through m^6^A sequencing analysis using the latest Illumina HiSeq sequencer. The results indicated that AZ leaded to alter expression profile in 198 m^6^A modification sites of lncRNAs (89 up-methylated and 109 down-methylated) which mainly enriched in lincRNAs. KEGG pathway analysis indicated that the “SNARE interactions in vesicular transport”, “GnRH signaling pathway”and “NOD-like receptor signaling pathway” may be closely associated with abnormal testis development of *X. laevis* due to exposure to AZ. Analysis results showed a negative correlation between m^6^A modification of lncRNA and lncRNA abundance, suggesting a regulatory role of m^6^A of lncRNAs in amphibious gene expression. Our study provides a fundamental contribution to possible molecular mechanisms underlying the reproductive system toxicity of AZ on male *X. laevis*.

However, in our study the first m^6^A transcriptome-wide map of lncRNAs of an amphibian species *X. laevis* presented here provides a starting roadmap for uncovering the role of m^6^A modification of lncRNAs that may affect/control amphibian testis development in the future. Meanwhile, our study characterized the differential m^6^A methylome of lncRNAs in the testis of *X. laevis* exposed to 100 μg/L AZ relative to the controls. The results suggested a possible association between m^6^A methylation and the regulation of developmental metabolism in the testis of *X. laevis* exposed to 100 μg/L AZ. So these results may provide a fundamental contribution to future studies aimed to gain deeper insights.

## Supplementary Information

**Additional file 1: Table S1.** The up and down methylated peaks.

## Data Availability

All data generated and or analyzed during this study are included in this published article and its supplementary information files.

## Abbreviations

M^6^A: N^6^-methyladenosine
*X. laevis*: *Xenopus laevis*
AZ: Atrazine
LncRNAs: Long non-coding RNAs
EDCs: Endocrine disrupting chemicals
FC: Fold change
KEGG: Kyoto Encyclopedia of Genes and Genomes
LincRNAs: Large intergenic noncoding RNAs
ceRNA: Competing endogenous RNA
SNARE: Soluble N-ethylmaleimide-sensitive factor attachment protein receptor
GnRH: GONADOTROPIN-releasing hormone
FSH: Follicle-stimulating hormone
LH: Luteinizing hormone
OX1R: Orexin receptors type 1
