# Supplementary material for: Comprehensive analysis of differences of N6-methyladenosine of lncRNAs between atrazine-induced and normal Xenopus laevis testis
Source: Genes Environ. 2021 Nov 6;43:49. doi: 10.1186/s41021-021-00223-0 (PMC8572474; doi:10.1186/s41021-021-00223-0)
Supplement: Supplementary file 1 — Additional file 1: Table S1. The up and down methylated peaks. [file 41021_2021_223_MOESM1_ESM.doc]

Table S1 The up and down methylated peaks

| Chromosome | txStart | txEnd | regulation | lncRNA | Fold change |
| --- | --- | --- | --- | --- | --- |
| NC_030726.1 | 167623498 | 167623720 | up | LOC108707576 | 143.6 |
| NC_030729.1 | 96905501 | 96905826 | up | LOC108713215 | 140.5 |
| NC_030729.1 | 42037587 | 42037701 | up | LOC108712839 | 119.9 |
| NC_030737.1 | 37948621 | 37948839 | up | LOC108697950 | 107.9 |
| NC_030726.1 | 78976541 | 78976920 | up | LOC108708210 | 101.9 |
| NC_030730.1 | 122514814 | 122514835 | up | LOC108713571 | 99.0 |
| NC_030727.1 | 93713360 | 93713391 | up | LOC108708955 | 96.1 |
| NC_030727.1 | 83393506 | 83393693 | up | LOC108708939 | 90.1 |
| NC_030724.1 | 53570461 | 53570705 | up | LOC108699628 | 87.1 |
| NC_030730.1 | 105478624 | 105478680 | up | LOC108713666 | 87.0 |
| NC_030728.1 | 5795854 | 5796160 | up | LOC108710760 | 81.1 |
| NC_030729.1 | 116070481 | 116070531 | up | LOC108713294 | 78.3 |
| NC_030725.1 | 121325613 | 121325700 | up | LOC108705073 | 75.1 |
| NC_030740.1 | 17585321 | 17586043 | up | LOC108702038 | 69.3 |
| NC_030740.1 | 112589281 | 112589660 | up | LOC108701691 | 66.4 |
| NC_030725.1 | 136561305 | 136561340 | up | LOC108707175 | 66.4 |
| NC_030734.1 | 85374361 | 85374560 | up | LOC108718732 | 63.4 |
| NC_030738.1 | 691601 | 691800 | up | LOC108698097 | 60.4 |
| NC_030726.1 | 159574941 | 159574999 | up | LOC108708713 | 57.4 |
| NC_030731.1 | 32497331 | 32497391 | up | LOC108714964 | 57.4 |
| NC_030726.1 | 36968666 | 36968800 | up | LOC108707976 | 54.5 |
| NC_030732.1 | 68099561 | 68100080 | up | LOC108716776 | 54.4 |
| NC_030728.1 | 4042701 | 4043000 | up | LOC108710733 | 54.3 |
| NC_030740.1 | 13565421 | 13565620 | up | LOC108700916 | 51.5 |
| NC_030729.1 | 37199467 | 37199491 | up | LOC108712306 | 48.5 |
| NC_030730.1 | 16262403 | 16262700 | up | LOC108713606 | 48.5 |
| NC_030738.1 | 34076181 | 34076740 | up | LOC108698909 | 48.5 |
| NC_030727.1 | 91555107 | 91555240 | up | LOC108709691 | 45.6 |
| NC_030732.1 | 153257327 | 153257700 | up | LOC108716388 | 45.5 |
| NC_030731.1 | 29238461 | 29238656 | up | LOC108715365 | 42.6 |
| NC_030735.1 | 36089361 | 36089840 | up | LOC108719949 | 42.6 |
| NC_030737.1 | 40417728 | 40417800 | up | LOC108697952 | 42.6 |
| NC_030729.1 | 23481861 | 23481944 | up | LOC108712706 | 42.6 |
| NC_030733.1 | 112466741 | 112467009 | up | LOC108717594 | 42.5 |
| NC_030737.1 | 24861701 | 24862240 | up | LOC108697198 | 30.9 |
| NC_030737.1 | 24885481 | 24886220 | up | LOC108697200 | 26.8 |
| NC_030738.1 | 30341561 | 30342560 | up | LOC108698406 | 19.8 |
| NC_030737.1 | 34102533 | 34102633 | up | LOC108697513 | 19.1 |
| NC_030724.1 | 88866521 | 88866560 | up | LOC108712203 | 18.6 |
| NC_030734.1 | 94911303 | 94911382 | up | LOC108718536 | 17.5 |
| NC_030738.1 | 4065222 | 4065560 | up | LOC108698186 | 12.9 |
| NC_030728.1 | 5797681 | 5798120 | up | LOC108710760 | 12.9 |
| NC_030740.1 | 45796094 | 45796184 | up | LOC108701248 | 12.0 |
| NC_030730.1 | 66264378 | 66264554 | up | LOC108714173 | 10.6 |
| NC_030741.1 | 32240972 | 32241332 | up | LOC108702789 | 10.4 |
| NC_030739.1 | 48704041 | 48704600 | up | LOC108700061 | 10.2 |
| NC_030739.1 | 47335621 | 47335769 | up | LOC108700052 | 9.4 |
| NC_030727.1 | 152870881 | 152871372 | up | LOC108709147 | 9.0 |
| NC_030728.1 | 138634341 | 138635100 | up | LOC108711775 | 8.8 |
| NC_030728.1 | 143435226 | 143435325 | up | LOC108710537 | 8.4 |
| NC_030727.1 | 116827421 | 116827940 | up | LOC108709799 | 8.1 |
| NC_030739.1 | 32016479 | 32016620 | up | med27.S | 8.0 |
| NC_030735.1 | 50393264 | 50393326 | up | LOC108720008 | 7.7 |
| NC_030740.1 | 73314624 | 73314688 | up | LOC108701855 | 7.4 |
| NC_030735.1 | 1612501 | 1613000 | up | LOC108695505 | 7.4 |
| NC_030734.1 | 85372521 | 85372556 | up | LOC108718732 | 7.3 |
| NC_030727.1 | 106921523 | 106922180 | up | LOC108709763 | 7.3 |
| NC_030736.1 | 49387482 | 49387631 | up | LOC108696987 | 7.2 |
| NC_030741.1 | 27775558 | 27775728 | up | LOC108702748 | 7.0 |
| NC_030739.1 | 26120861 | 26121500 | up | LOC108700634 | 6.9 |
| NC_030732.1 | 67044456 | 67044760 | up | LOC108716773 | 6.8 |
| NC_030725.1 | 54792540 | 54792740 | up | LOC108706697 | 6.8 |
| NC_030727.1 | 47831537 | 47831803 | up | LOC108709428 | 6.8 |
| NC_030731.1 | 22223961 | 22224139 | up | LOC108714946 | 6.6 |
| NC_030724.1 | 12829121 | 12829247 | up | LOC108696324 | 6.4 |
| NC_030740.1 | 16602835 | 16602940 | up | LOC108701994 | 6.0 |
| NC_030725.1 | 119894993 | 119895000 | up | LOC108707074 | 6.0 |
| NC_030727.1 | 143487370 | 143487475 | up | LOC108709021 | 5.8 |
| NC_030724.1 | 152923293 | 152923440 | up | LOC495492.L | 5.6 |
| NC_030732.1 | 154664461 | 154664674 | up | LOC108716391 | 5.6 |
| NC_030731.1 | 26416761 | 26417620 | up | LOC108715348 | 5.5 |
| NC_030732.1 | 63834871 | 63834960 | up | LOC108716758 | 5.4 |
| NC_030741.1 | 35583841 | 35584180 | up | LOC108702834 | 5.2 |
| NC_030729.1 | 94009015 | 94009040 | up | LOC108712383 | 5.1 |
| NC_030733.1 | 51537561 | 51538440 | up | LOC108717899 | 5.1 |
| NC_030726.1 | 149778456 | 149778555 | up | LOC108707745 | 5.1 |
| NC_030724.1 | 128869219 | 128869640 | up | LOC108714507 | 5.0 |
| NC_030741.1 | 101914761 | 101915244 | up | LOC108703267 | 5.0 |
| NC_030734.1 | 38446523 | 38446605 | up | LOC108718881 | 5.0 |
| NC_030739.1 | 87922621 | 87922986 | up | LOC108700591 | 5.0 |
| NC_030724.1 | 217939681 | 217940120 | up | LOC108719063 | 5.0 |
| NC_030725.1 | 103843125 | 103843188 | up | LOC108706955 | 4.8 |
| NC_030733.1 | 90924658 | 90924901 | up | LOC108717576 | 4.8 |
| NC_030726.1 | 172955181 | 172955316 | up | LOC108708780 | 4.7 |
| NC_030740.1 | 42197528 | 42197554 | up | LOC108701190 | 4.7 |
| NC_030725.1 | 67176881 | 67177247 | up | LOC108704462 | 4.4 |
| NC_030725.1 | 119895081 | 119895580 | up | LOC108707074 | 4.3 |
| NC_030724.1 | 86739629 | 86739868 | up | LOC108711999 | 4.2 |
| NC_030727.1 | 37459216 | 37459407 | up | LOC108709366 | 4.0 |
| NC_030730.1 | 56714441 | 56715000 | down | LOC108713510 | 108.0 |
| NC_030737.1 | 3748163 | 3748254 | down | LOC108697148 | 93.6 |
| NC_030727.1 | 30334141 | 30334313 | down | LOC108709317 | 82.1 |
| NC_030727.1 | 141936052 | 141936238 | down | mmp8.S | 79.6 |
| NC_030741.1 | 23682418 | 23682540 | down | LOC108702712 | 77.2 |
| NC_030731.1 | 10726561 | 10726740 | down | LOC108715064 | 77.2 |
| NC_030733.1 | 59942967 | 59943104 | down | LOC108717426 | 72.3 |
| NC_030725.1 | 4717538 | 4717900 | down | LOC108706487 | 71.8 |
| NC_030736.1 | 79214125 | 79214280 | down | LOC108695842 | 71.8 |
| NC_030736.1 | 43073001 | 43073560 | down | LOC108696274 | 70.0 |
| NC_030734.1 | 75910758 | 75910851 | down | LOC108718516 | 70.0 |
| NC_030733.1 | 112284733 | 112285200 | down | LOC108718211 | 70.0 |
| NC_030733.1 | 112242581 | 112243040 | down | LOC108718210 | 70.0 |
| NC_030733.1 | 112285381 | 112285920 | down | LOC108718211 | 68.8 |
| NC_030728.1 | 2434501 | 2434514 | down | LOC108710718 | 63.9 |
| NC_030725.1 | 166877027 | 166877106 | down | LOC108707314 | 63.9 |
| NC_030741.1 | 30359125 | 30359218 | down | LOC108702385 | 63.9 |
| NC_030740.1 | 30131577 | 30131749 | down | LOC108701037 | 63.9 |
| NC_030733.1 | 110418161 | 110418700 | down | LOC108717477 | 63.9 |
| NC_030726.1 | 44470561 | 44470652 | down | LOC108707455 | 62.1 |
| NC_030740.1 | 3462001 | 3462027 | down | LOC108700803 | 62.1 |
| NC_030739.1 | 97268541 | 97268831 | down | LOC108700423 | 60.3 |
| NC_030732.1 | 154726247 | 154726430 | down | LOC108717257 | 57.3 |
| NC_030727.1 | 29107641 | 29108180 | down | LOC108708877 | 56.6 |
| NC_030733.1 | 68350177 | 68350295 | down | LOC108717562 | 56.6 |
| NC_030725.1 | 48694941 | 48695045 | down | LOC108704374 | 55.4 |
| NC_030738.1 | 21813296 | 21813320 | down | LOC108698327 | 55.4 |
| NC_030726.1 | 122722961 | 122723180 | down | LOC108708488 | 53.6 |
| NC_030726.1 | 46320723 | 46321180 | down | LOC108708034 | 53.6 |
| NC_030737.1 | 55354715 | 55354780 | down | LOC108697237 | 53.0 |
| NC_030725.1 | 125121581 | 125121772 | down | LOC108707102 | 50.6 |
| NC_030725.1 | 78624841 | 78624875 | down | LOC108704619 | 50.6 |
| NC_030740.1 | 15058727 | 15058900 | down | LOC108700930 | 50.6 |
| NC_030738.1 | 111332852 | 111333000 | down | LOC108699420 | 50.6 |
| NC_030735.1 | 68590030 | 68590140 | down | LOC108720101 | 50.6 |
| NC_030735.1 | 19122485 | 19122500 | down | LOC108719668 | 50.6 |
| NC_030724.1 | 117844733 | 117844840 | down | LOC108713877 | 50.6 |
| NC_030729.1 | 27169241 | 27169640 | down | LOC108712756 | 50.6 |
| NC_030738.1 | 9665381 | 9665820 | down | LOC108698231 | 50.0 |
| NC_030737.1 | 87072381 | 87072801 | down | LOC108697999 | 50.0 |
| NC_030740.1 | 18046621 | 18047300 | down | LOC108702046 | 48.8 |
| NC_030724.1 | 77261201 | 77261524 | down | LOC108719654 | 47.0 |
| NC_030727.1 | 103955381 | 103955482 | down | LOC108708980 | 47.0 |
| NC_030724.1 | 122003780 | 122003900 | down | LOC108714006 | 47.0 |
| NC_030736.1 | 49188401 | 49188670 | down | LOC108696986 | 45.8 |
| NC_030725.1 | 178948806 | 178948980 | down | LOC108707360 | 44.0 |
| NC_030730.1 | 62626357 | 62626640 | down | LOC108714135 | 43.3 |
| NC_030725.1 | 172084933 | 172085160 | down | LOC108705454 | 42.1 |
| NC_030740.1 | 112533541 | 112533580 | down | LOC108701690 | 42.1 |
| NC_030725.1 | 6592061 | 6592193 | down | LOC108706496 | 42.1 |
| NC_030737.1 | 40784081 | 40784620 | down | LOC108697223 | 42.1 |
| NC_030733.1 | 118973541 | 118973596 | down | LOC108717600 | 42.1 |
| NC_030736.1 | 7800696 | 7800866 | down | LOC108696061 | 40.3 |
| NC_030733.1 | 95310401 | 95310434 | down | LOC108717456 | 24.2 |
| NC_030724.1 | 154796883 | 154797045 | down | LOC108716055 | 22.0 |
| NC_030733.1 | 51580145 | 51580186 | down | LOC108717899 | 17.8 |
| NC_030736.1 | 104984601 | 104985160 | down | LOC108695900 | 16.1 |
| NC_030727.1 | 46523405 | 46523800 | down | LOC108709421 | 16.0 |
| NC_030734.1 | 123459741 | 123460021 | down | LOC108718827 | 15.7 |
| NC_030736.1 | 54688208 | 54688578 | down | LOC108696319 | 14.0 |
| NC_030731.1 | 28754961 | 28755180 | down | LOC108715361 | 14.0 |
| NC_030729.1 | 19325961 | 19326279 | down | LOC108712656 | 13.2 |
| NC_030725.1 | 40657971 | 40658185 | down | LOC108704322 | 12.1 |
| NC_030727.1 | 92185182 | 92185235 | down | LOC108709696 | 11.9 |
| NC_030738.1 | 122543 | 122900 | down | LOC108698078 | 10.8 |
| NC_030726.1 | 83192641 | 83192705 | down | LOC108707503 | 10.2 |
| NC_030741.1 | 26729353 | 26729396 | down | LOC108702738 | 10.0 |
| NC_030741.1 | 17511101 | 17511176 | down | LOC108702685 | 9.7 |
| NC_030727.1 | 121329572 | 121330020 | down | LOC108708993 | 9.7 |
| NC_030726.1 | 101597088 | 101597093 | down | LOC108707521 | 9.4 |
| NC_030741.1 | 105661 | 105883 | down | LOC108702451 | 9.0 |
| NC_030734.1 | 35415031 | 35415071 | down | LOC108718861 | 8.7 |
| NC_030726.1 | 8391990 | 8392137 | down | LOC108707844 | 8.4 |
| NC_030734.1 | 102707230 | 102707440 | down | LOC108719183 | 8.1 |
| NC_030725.1 | 99344090 | 99344327 | down | LOC108704829 | 8.1 |
| NC_030734.1 | 35415964 | 35416010 | down | LOC108718861 | 7.9 |
| NC_030729.1 | 63991221 | 63991748 | down | LOC108712343 | 7.9 |
| NC_030732.1 | 70373621 | 70373659 | down | LOC108716788 | 7.6 |
| NC_030740.1 | 57041687 | 57041863 | down | LOC108701319 | 7.2 |
| NC_030729.1 | 1581529 | 1581886 | down | LOC108712440 | 7.0 |
| NC_030730.1 | 122531988 | 122532093 | down | LOC108713571 | 6.9 |
| NC_030737.1 | 89038701 | 89038704 | down | LOC108697907 | 6.9 |
| NC_030727.1 | 54014462 | 54014618 | down | LOC108709445 | 6.9 |
| NC_030730.1 | 64405361 | 64405583 | down | LOC108714152 | 6.8 |
| NC_030735.1 | 126774273 | 126774949 | down | LOC108695469 | 6.7 |
| NC_030738.1 | 690954 | 691105 | down | LOC108698097 | 6.6 |
| NC_030731.1 | 40183587 | 40184623 | down | LOC108715416 | 6.4 |
| NC_030728.1 | 6692766 | 6692918 | down | LOC108710767 | 6.3 |
| NC_030731.1 | 63407605 | 63407780 | down | LOC108715095 | 6.1 |
| NC_030729.1 | 25179381 | 25179545 | down | LOC108712724 | 6.1 |
| NC_030729.1 | 1580121 | 1580373 | down | LOC108712440 | 5.7 |
| NC_030740.1 | 53190718 | 53191100 | down | LOC108701311 | 5.7 |
| NC_030724.1 | 93648915 | 93649000 | down | LOC108712235 | 5.5 |
| NC_030731.1 | 15784085 | 15784533 | down | LOC108715265 | 5.4 |
| NC_030741.1 | 41805521 | 41806124 | down | LOC108702892 | 5.4 |
| NC_030741.1 | 39004121 | 39004285 | down | LOC108702230 | 5.2 |
| NC_030726.1 | 61944589 | 61944614 | down | LOC108707487 | 5.1 |
| NC_030732.1 | 139703770 | 139703820 | down | LOC108716379 | 5.1 |
| NC_030727.1 | 56285981 | 56286720 | down | LOC108708913 | 4.9 |
| NC_030730.1 | 25634781 | 25634826 | down | LOC108713913 | 4.8 |
| NC_030734.1 | 74947245 | 74947560 | down | LOC108718690 | 4.8 |
| NC_030733.1 | 59734861 | 59735007 | down | LOC108717938 | 4.7 |
| NC_030724.1 | 190232021 | 190232146 | down | LOC108718074 | 4.4 |
| NC_030738.1 | 20827721 | 20828174 | down | LOC108698309 | 4.4 |
| NC_030741.1 | 33288656 | 33288866 | down | LOC108702813 | 4.3 |
| NC_030733.1 | 61519101 | 61519204 | down | LOC108717949 | 4.2 |
| NC_030740.1 | 45809437 | 45809972 | down | LOC108701248 | 4.2 |
| NC_030738.1 | 65306446 | 65306537 | down | LOC108698726 | 4.1 |
| NC_030737.1 | 21903196 | 21903406 | down | LOC108697190 | 4.1 |

txStart/txEnd: Start/end position of the differentially methylated RNA peak.
